# ﻿*Amanoacondorensis* (Phyllanthaceae), a new shrubby species from the Cordillera del Condor in southern Ecuador

**DOI:** 10.3897/phytokeys.227.104703

**Published:** 2023-06-02

**Authors:** John L. Clark, David A. Neill

**Affiliations:** 1 Marie Selby Botanical Gardens, 1534 Mound St., Sarasota, FL 34236, USA Marie Selby Botanical Gardens Sarasota United States of America; 2 Universidad Estatal Amazónica, Puyo, Pastaza, Ecuador Universidad Estatal Amazónica Puyo Ecuador

**Keywords:** Andean tepui, Andes, androphore, Ecuador, Nangaritza Plateau, Phyllanthaceae, taxonomy

## Abstract

A new species of *Amanoa* (Phyllanthaceae) is described from the sandstone Nangaritza Plateau in the Cordillera del Cóndor Region in southern Ecuador. *Amanoacondorensis* J.L.Clark & D.A.Neill is a small tree, 4 m tall that is only known from the type collection. The new species is distinct by a shrub habit, presence of coriaceous leaves with an acuminate apex, and congested inflorescences. The relatively high elevation of the type locality, presence of an androphore, and the habit as shrub or low tree are an unusual combination for *Amanoa*. The conservation status of *A.condorensis* is assessed as Critically Endangered (CR), based on IUCN Criteria.

## Abstract (es)

Se describe una nueva especie de *Amanoa* (Phyllanthaceae) de la meseta de arenisca de Nangaritza en la región de la Cordillera del Cóndor en el sur de Ecuador. *Amanoacondorensis* J.L.Clark & D.A.Neill es un pequeño árbol de 4 metros de altura que sólo se conoce de la colección tipo. La nueva especie se distingue por la presencia de inflorescencias congestionadas, hojas coriáceas con ápice acuminado, y porte arbustivo. La elevación relativamente más alta de la localidad tipo, la presencia de un andróforo, y el hábito de arbusto o árbol pequeño son una combinación inusual para *Amanoa*. El estado de conservación de *A.condorensis* se evalúa como En Peligro Crítico (CR) según los Criterios de la UICN.

## ﻿Introduction

*Amanoa* Aublet is a genus with a trans-Atlantic disjunct distribution. Most species of *Amanoa* are endemic to the Neotropics (14 species presently known: Ulloa Ulloa et al. (2018)) and two species are endemic to Africa. The most recent monograph is by Pax and Hoffman (1922) and, at that time, *Amanoa* comprised nine species. An overview of the Neotropical members of *Amanoa* was provided by Hayden (1990), who summarized and evaluated all currently-recognized taxa from the Neotropics. Hayden (1990) designated taxonomic typifications, updated circumscriptions with synonyms, and described four new species. An additional endemic species to Pará State of Brazil was discovered and described by Secco (2014) and an update to the Brazilian species of *Amanoa*, with detailed descriptions and illustrations, was provided by Secco et al. (2014). This included the resurrection of *A.pubescens* Steyerm. which was later placed in synonymy with *A.almerindae* Leal (Secco and Rosário 2016). The description of *Amanoacondorensis* brings the number of *Amanoa* to 17 species, of which 15 are Neotropical in distribution.

Phylogenetic studies (Wurdack et al. 2004; Hoffmann et al. 2006) support the placement of *Amanoa* in Phyllanthaceae Martynov subfamily Phyllanthoideae, tribe Brideliae Müll. Arg., subtribe Amanoinae Pax & K. Hoffm. *Amanoa* is the only genus currently recognized in subtribe Amanoinae. A detailed overview of the morphological characters that define *Amanoa* was summarized by Hayden (1999). Several field-based vegetative and reproductive features help identify *Amanoa*, such as the presence of two-ranked leaves and intrapetiolar stipules. Other vegetative characters that *Amanoa* species share include coriaceous leaves that are evergreen, glabrous and with entire margins. The flower morphology for the Neotropical members of *Amanoa* is remarkable for the presence of an extra-staminal nectary and sessile stigmas (Hayden 1990). The presence of a short androphore is sometimes present. The flowers usually consist of showy sepals and highly-reduced petals that are difficult to see without a 10× hand lens.

## ﻿Materials and methods

Plants were vouchered and photographed during a 2017 field expedition to Ecuador. Specimens were deposited at the Universidad Estatal Amazónica (**ECUAMZ**), Marie Selby Botanical Gardens (**SEL**) and the Smithsonian Institution’s National Museum of Natural History (**US**). Digital images were taken of live specimens in the field using a Nikon D100 DSLR with a Nikon 105 mm lens and a Nikon SB-29s ring flash. Morphological observations and measurements were made from live collections and herbarium specimens.

We assessed the extinction risk of *Amanoacondorensis* following the IUCN (2012) and guidelines of the IUCN Standards and Petitions Committee (2022). We considered observations, collection localities, and population estimates from fieldwork. Species extent of occurrence (EOO) and area of occupancy (AOO) were calculated using *GeoCAT* (Bachman et al. 2011; http://geocat.kew.org/) with the default setting of 2 km^2^ grid.

## ﻿Taxonomic treatment

### ﻿ Amanoa condorensis

Taxon classificationPlantaeMalpighialesPhyllanthaceae

J.L.Clark & D.A.Neill sp. nov.

62ECEB17-CDCC-53FE-ABEC-C5903A3C8613

urn:lsid:ipni.org:names:77320398-1

Figs 1 , 2

#### Diagnosis.

Amongst the Neotropical species of *Amanoa*, *A.condorensis* shares with *A.almerindae* Leal. and *A.caribaea* Krug & Urb. the presence of an androphore, formed from the fusion of the basal filaments. Differs from *Amanoaalmerindae* by the densely pubescent and more widely-spaced flowers along an inflorescence axis 5.5–11 cm long in *A.almerindae* vs. nearly glabrous inflorescence axis to 4.5 cm long in *A.condorensis.* Differs from *A.caribaea* by the presence of inflorescences in an elongate erect raceme appearing congested throughout vs. inflorescences in elongate erect spikes with evenly-spaced fascicles (i.e. not congested throughout) in *A.caribaea*.

#### Type.

**Ecuador. Zamora-Chinchipe**: Nangaritza Cantón, Cordillera del Cóndor, trail west of Cabañas Yankuam in conservation area that is owned/operated by ATASMO (Asociación de Trabajadores Autónomos San Miguel de las Orquídeas). Forested tepui (sandstone plateau). Summit ridge and sandstone cliff face. Low scrub and elfin forest, canopy mostly 2–3 m tall, occasional emergent trees to 5 m, 4°15'47.5"S, 78°41'28.1"W, 1840 m elev., 10 Mar 2017, *J.L. Clark, J.A. Mayr & D.A. Neill 15257* (holotype: ECUAMZ [08582]; isotypes: SEL [120063], US).

#### Description.

Tree, 4 m tall. ***Leaves*** 1.2–2.5 × 3–4.5 cm, oblong to ovate, coriaceous, glabrous, secondary venation suppressed adaxially when live and becoming prominent when dry, abaxial secondary venation prominent when live and dry, sparsely pubescent on abaxial and adaxial surface, blade flat, base narrowly cuneate to acute, apex acute; petiole 4–8 mm long, slender, black, rugose, sparsely pilose, sometimes appearing sessile from the intrapetiolar stipule; stipules conspicuous, to 0.5 mm long, triangular and glabrous. ***Inflorescence*** terminal or lateral, to 4.5 cm long, in congested erect elongate raceme-like inflorescences derived from reduced cymules, the primary inflorescence branch dark black, nearly glabrous to sparsely pubescent with short curved trichomes of 3–5 cells, each flower or pair of flowers subtended by a prominent brown triangular bracteole, 2–3 mm long and glabrous. ***Staminate flowers*** actinomorphic, 5-merous, 6.5–7.5 mm in diam. during anthesis, on short pedicels and appearing sessile; sepals 3.0–3.5 × 1.5–2.0 mm, broadly ovate, glabrous, white; petals ca. 1 × 1.5 mm, reniform, margins entire, glabrous; androecium surrounded by a prominent hypogynous extra-staminal disc, stamens fused at base, forming a short androphore, filaments 1.5–2 mm long, anthers 1–2 mm long, dehiscing longitudinally; pistillode ca. 2.5 mm long with trilobed apex, mature gynoecium or pistillate flowers not observed. Fruits not observed.

#### Phenology.

Mature male flowers were observed in March.

#### Etymology.

The specific epithet is derived from the Cordillera del Cóndor mountain range in southern Ecuador where this species is presumably endemic.

#### Distribution and associated vegetation.

*Amanoacondorensis* is only known from the type collection. It is presumed endemic to the Cordillera del Condór from where it was collected during a collaborative field course in 2017 with the Lawrenceville School (Lawrenceville, NJ, USA) and the Universidad Estatal Amazónica (Puyo, Ecuador). The type locality is situated at the summit ridge of a sloping plateau (1840 m elevation) west of the upper Nangaritza River, with nutrient-poor soil derived from the Cretaceous Hollín sandstone formation. The vegetation at the site is a low, dense scrub, dominated by shrubs and low trees 3–5 m high. These environments in the Cordillera del Cóndor and other mountain ranges east of the main Andean chain in Ecuador and Peru have been referred to as “Andean tepuis” in recognition of the similarity in vegetation and some phytogeographic connections with the low-nutrient sandstone tepuis of the Guiana Shield Region (Neill et al. 2014; Neill 2018). Associated plants at the collection site include the local endemic *Blakeanangaritzana* D.Fernández, C.Ulloa & Penneys (Melastomataceae) as well as other species that are common in Andean tepui elfin forest vegetation at 1500–2000 m elevation, including *Godoyaobovata* Ruiz & Pav. (Ochnaceae), *Ternstroemiacircumscissilis* Kobuski (Pentaphylacaceae), *Macrocarpaeainnarrabilis* J.R.Grant (Gentianaceae), *M.ericii* J.R.Grant (Gentianaceae), *Cybianthusmagnus* (Mez) Pipoly (Primulaceae), *Podocarpustepuiensis* J.Buchholz & N.E. Gray (Podocarpaceae), *Ladenbergiafranciscana* C.M.Taylor (Rubiaceae), *Pterozoniumbrevifrons* (A.C.Sm.) Lellinger (Pteridaceae) and *Everardiamontana* Ridl. (Cyperaceae).

#### Preliminary assessment of conservation status.

The type locality is located within a community-based protected forest managed by the Asociación de Trabajadores Autónomos San Miguel de las Orquídeas (ATASMO) on the remote summit ridge of a sloping Andean tepui at 1840 m elevation, accessible via a 6 km trail from the Cabañas Yankuam (= Yankuam Lodge) located at 890 m elevation on the banks of the Río Nangaritza. Yankuam is a family-owned lodge specializing in bird tours and ecotourism. The Río Nangaritza is at risk from ongoing illegal mining. Many of the areas visited in 2017 (especially along the Río Nangaritza) have been invaded by illegal mining operations. The forest that corresponds to the only known population of *Amanoacondorensis* is protected by the local community, which was granted this jurisdiction by the Ministry of Environment of Ecuador. Between 2017 and 2019 (three years), annual field courses were run with Cabañas Yankuam and the local community to different sandstone tepuis. *Amanoacondorensis* was only observed once throughout three consecutive years of field courses. Following the IUCN (2012) and guidelines of the IUCN Standards and Petitions Committee (2022), *Amanoacondorensis* is categorized as Critically Endangered (CR), based on the following criteria: B1, B2 ab (all criteria), area of occupancy (AOO) is calculated at 4 km^2^ (criterion B2 < 10 km^2^). The species is known only from the type collection; it is hoped that this publication will stimulate more intensive search for additional material near the type locality and on other Andean tepuis in the region, including specimens with pistillate flowers.

#### Comments.

Most species of *Amanoa* are canopy to sub-canopy trees in the lowland tropics. The only other species that is known to occur above 1000 m is *Amanoasteyermarkii* Jabl. in the riparian and tepui forests of the Venezuelan Guayana Region at 1500–2100 m elevation (Hayden 1999). *Amanoacondorensis* was collected above 1800 m. *Amanoacondorensis* differs from *A.steyermarkii* by its sessile staminate flowers (vs. pedicellate flowers in *A.steyermarkii*) and its congested inflorescence (vs. the relatively lax inflorescence in *A.steyermarkii*). It also differs in its shrubby habit (vs. trees in *A.steyermarkii*).

*Amanoacondorensis* differs from most other congeners in its relatively small height of 4 m. Most other *Amanoa* species are understory to emergent canopy trees above 12 m tall (e.g. *A.guianensis* Aubl. reaches 35 m tall). It is also distinguished by the relatively small, thick and sclerophyllous leaves, characters that are shared by many plants in different genera that occur in the dense scrub vegetation of the Andean tepuis (Neill et al. 2014) and that may be an adaptation to the nutrient-poor sandy soils of these habitats. The two other *Amanoa* species that are recorded as shrubs or small trees include *A.cupatensis* Huber and *A.almerindae*; both species are endemic to the white-sand areas of the Rio Orinoco-Casiquiare lowlands and the adjacent Río Negro lowlands in Amazonas State of Venezuela and Amazonas State of Brazil. *Amanoacupatensis* is a low shrub less than 2 m tall. *Amanoacondorensis* differs from *A.cupatensis* by the presence of an acute leaf apex (vs. rounded to emarginate leaf apex in *A.cupatensis*).

**Figure 1.**
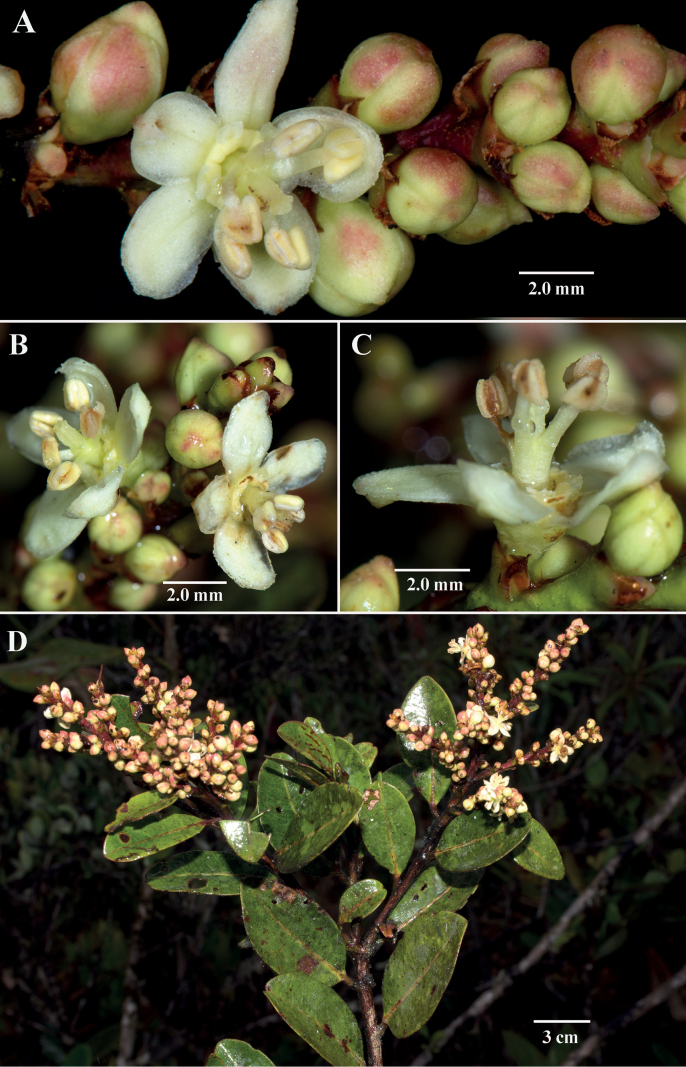
*Amanoacondorensis* J.L.Clark & D.A.Neill **A–C** mature staminate flower featuring prominent sepals, reduced petals, extra-staminal disc and androphore **D** shoot featuring two-ranked foliage and congested inflorescences. (**A–D***J.L. Clark*, *D.A. Neill & J. Mayr 15257*). Photos by John L. Clark.

*Amanoacondorensis* shares the following characters with other congeners: an extra-staminal disc (Fig. 1), intrapetiolar stipules, and a showy perianth represented by conspicuous sepals and inconspicuous petals (Fig. 1). The petals are only apparent with a 10× hand lens or higher magnification (Fig. 1A). The intrapetiolar stipules in *A.condorensis* are conspicuous on herbarium specimens and in the field.

**Figure 2.**
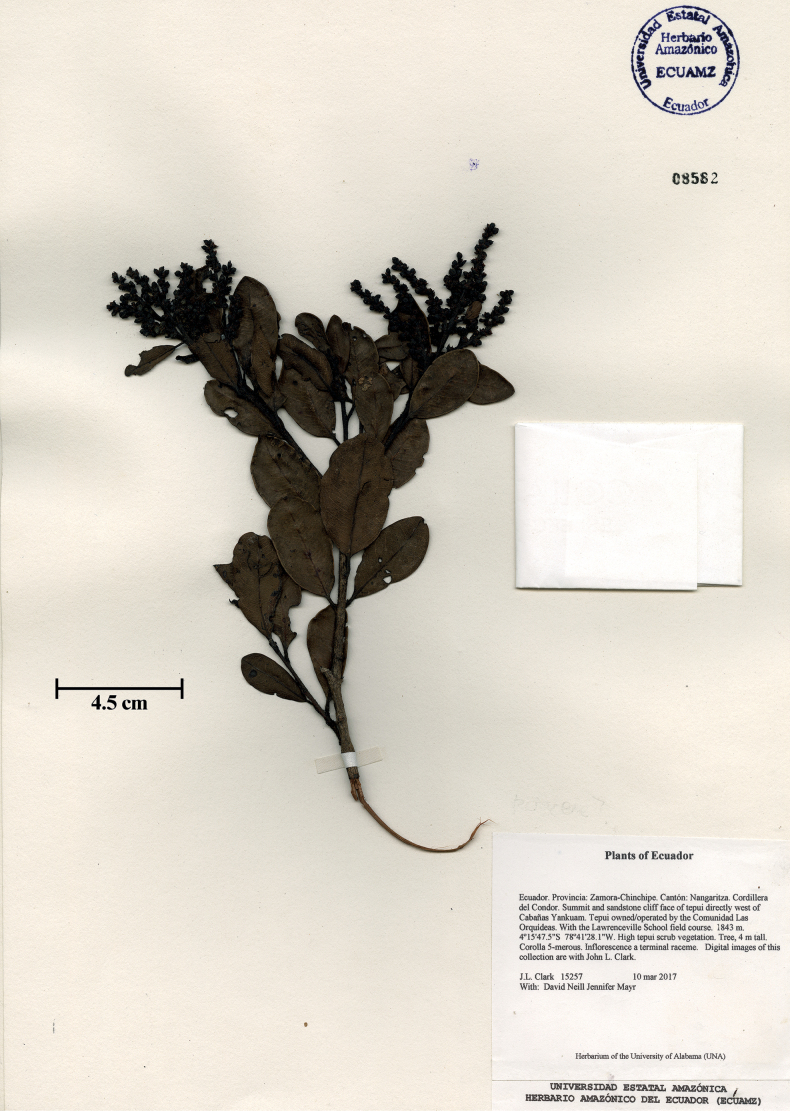
Holotype (ECUAMZ) of *Amanoacondorensis* J.L.Clark & D.A.Neill.

*Amanoacondorensis* has an androphore, a structure formed by the basal fusion of the filaments (Fig. 1C), a character that is only reported for two additional species: *A.almerindae* and *A.caribaea* Krug & Urb. The inflorescence of *A.caribaea* is an elongate spike-like inflorescence with broadly spaced fascicles of numerous flowers. In contrast, *A.condorensis* has congested inflorescences, but never more than two flowers per bracteole. *Amanoacaribaea* is endemic to the Caribbean (Dominica and Guadeloupe), where it is reported as a common co-dominant important timber tree in native forests of Dominica (Nicolson 1991).

*Amanoapubescens* was previously considered a synonym of *A.almerindae*, but was separated and resurrected by Secco et al. (2014), based on the presence of an androphore in *A.pubescens* and its absence in *A.almerindae*. However, Secco and Rosário (2016) re-examined the type material of both taxa and determined that *A.almerindae* does possess an androphore that is evident in open staminate flowers, but not in bud. As a result, Secco and Rosário (2016), once again, reduced *A.pubescens* to synonymy with *A.almerindae*. *Amanoaalmerindae* differs from *A.condorensis* in its thinner, larger more widely-spaced leaves, and more elongate inflorescences with more broadly spaced fascicles (vs. thick, sclerophylous, smaller leaves and short, congested inflorescence in *A.condorensis*).

Hayden (1990) reported that most flowers in *Amanoa* are protandrous and species can be either monoecious or dioecious. Only two Neotropical species are known to be dioecious: *A.glaucophylla* Müll.Arg of Amazonian and coastal Brazil, as well as Amazonian Colombia and Venezuela and *A.anomala* Little, endemic to the Pacific coast of Ecuador. The remaining species are presumed to be monoecious, but this has not been confirmed for most species. The flowers featured here (Fig. 1) have mature androecia with vestigial gynoecia. Therefore, if the species is monoecious, we expect the pistillate flowers would appear at a later stage. If dioecious, then separate staminate and pistillate individuals would be expected.

## Supplementary Material

XML Treatment for Amanoa condorensis

## ﻿Additional information

### Author ORCIDs

John L. Clark https://orcid.org/0000-0002-1414-6380

David A. Neill https://orcid.org/0000-0002-5143-9430

### Data availability

All of the data that support the findings of this study are available in the main text or Supplementary Information.
